# How Do Acculturation, Maternal Connectedness, and Mother-Daughter Sexual Communication Affect Asian American Daughters’ Sexual Initiation?

**DOI:** 10.31372/20200501.1080

**Published:** 2020

**Authors:** BoRam Kim, Yurun Cai, Teri Aronowitz

**Affiliations:** University of Massachusetts Boston, Boston, Massachusetts, United States

**Keywords:** acculturation, maternal connectedness, mother-daughter sexual communication, sexual initiation, Asian American adolescents

## Abstract

*Purpose:* There was a growth of approximately ten million Asian American individuals in the United States between 2000 and 2015. Asian Americans have conservative values surrounding sexual health and sexual communication is a cultural taboo. Researchers have shown discrepancies on whether the level of acculturation influences Asian mother-daughter sexual communication. In other minority populations there is evidence that a connected mother–daughter relationship increases sexual communication and delays sexual initiation. The purpose of this study was to examine whether mother-daughter connectedness and level of acculturation predict sexual communication in turn affecting the age of female Asian emerging adult’s sexual initiation. *Methods:* This was a longitudinal, secondary analysis of AddHealth examining whether mother-daughter connectedness and level of acculturation predict sexual communication. There were 243 Asian American mother-daughter dyads in Wave I with linked data in Wave III who were included in the study. Acculturation, connectedness, and sexual communication were all measured using interval level data. *Results:* Connectedness did not significantly contribute to the relationship between any of the concepts. Although it was predicted that sexual communication would delay initiation, the opposite was found. Also, communication mediated the relationship between acculturation and initiation. *Conclusions:* Further studies are needed to explore how connectedness is defined by Asian American mother-daughter dyads. In addition, more detailed operational definitions of acculturation and communication are needed, specifically the timing of sexual communication.

## Background

One of the fastest growing ethnic groups in the United States (U.S.) today is the Asian American (AA) population growing from 12 million in 2000 (U.S. Census Bureau, 2002) to 21 million in 2015 (U.S. Census Bureau, 2017). Young adults between the ages of 20 and 29 years makes up nearly 16% of the total AA population, which is the highest percentage of all AA age groups (U.S. Census Bureau, 2015). AAs are the largest growing group immigrating to the U.S., however, research focusing on their sexual health has been limited (U.S. Census Bureau, 2017).

Historically, there has been a myth that AAs are a model minority group in terms of practicing safe sexual behaviors (Wong, Lai, Nagasawa, & Lin, 1998). Despite this general societal assumption, AA youth do participate in risky behaviors such as inconsistent use of condoms and alcohol use with sexual activity (Zhao, Lau, Vermette, Liang, & Flores, 2017). AA emerging adults do not see themselves as being at risk for HIV (Hahm, Lee, Rough, & Strathdee, 2012), even though one in five AAs are infected with HIV (Centers for Disease Control and Prevention [CDC], 2018). As opposed to the decreasing HIV rates among African American (53.8/100,000 in 2010, 44.3/100,000 in 2015) and Caucasians (6.0/100,000 in 2010, 5.3/100,000 in 2015) (CDC, 2016), AAs have had a continuous increase in HIV infection rates from 30.0/100,000 in 2010 to 33.4/100,000 in 2015 (CDC, 2016). Although sexual minorities and gender expansive (nonbinary) youth may increase at risk for HIV, this study focuses on heterosexual relationships.

The risky sexual behaviors that AA youth participate in maybe closely associated with the degree of their acculturation. Acculturation is a concept used to define changes in individuals’ cultural values and practices, language, and behaviors as they adapt from their heritage culture to the mainstream society (Berry, 1997; Berry, Kim, Minde, & Mok, 1987). In regard to AAs living in the U.S., acculturation has been measured by length of time living in the U.S., language preference (e.g., English or non-English language), and preference to Asian or Non-Asian social communities (Hahm et al., 2012; Murray et al., 2014; Tong, 2013). There are several measures of acculturation used to assess AA’s adaptation to U.S. society (Ryder, Alden, & Paulhus, 2000; Suinn, Rickard-Figueroa, Lew, & Vigil, 1987). Many researchers only use length of stay in U.S. or language spoken. The Asian Value Scale has been developed to operationalize Asian values and the effect that the level of acculturation has on maintaining these values (Kim, Atkinson, & Yang, 1999). Employing this measure, researchers have found that the longer AAs live in the U.S., the more they are exposed and adapt to American society which is more open and less conservative about sexual health than Asian cultures (Tong, 2013).

Several studies have shown that the more acculturated and Americanized AA adolescents are the more likely they are to initiate sexual activity at an early age and participate in risky sexual behaviors (including alcohol use with sexual activity) than less acculturated AA adolescents (Hahm, Lahiff, & Barreto, 2006; Murray et al., 2014; Schwartz et al., 2011). For example, 30 percent of AA female adolescents who are in grades 7–12 and speak English at home reported they have already initiated sexual behaviors. This is compared to 10 percent of AA adolescents the same age who speak their heritage language at home (Asian & Pacific Islander American Health Forum, 2009; Hahm et al., 2006). Also, recent Asian immigrants to Canada (a country very similar culturally to the U.S.) have more conservative sexual attitudes than long-term Canadian immigrants or Canadian-born Asians (Meston, Trapnell, & Gorzalka, 1998). These conservative sexual attitudes are prominent among East and Southeast Asian families and continue to influence Asian families’ ability to openly discuss sexual health today (Lee et al., 2013; Zhao et al., 2017).

These studies reflect the relationship between acculturation and AAs’ sexual attitudes and behavior. Fifty-nine percent of U.S.-born AA youth who are highly acculturated reported that they have had sex, but nearly 50 percent of those AAs reported they did not use a condom during recent vaginal or anal sex (Hahm et al., 2012). While acculturated AAs are practicing unsafe sexual behaviors, they maintain a strong cultural stigma of avoiding open communication about sexual health topics. Secondary to the general Asian conservative culture and social norm surrounding sexual health (Zhao et al., 2017), AA youth currently continue to refrain from discussing these topics with family and partners. In one subset of Asian families, Filipino American adolescents who are more acculturated are less likely to have open sexual communication with their mothers (Chung et al., 2007).

Because value transmission from the mother to daughter occurs within a relationship (Vygotsky, 1978), the importance of facilitating a connected relationship between mother and daughter is critical. The closer daughters are to their mothers the more open their sexual communication (Aronowitz & Agbeshie, 2012). Since mothers are the primary sex educator of daughters, it has been shown that increasing connectedness is critical to increasing open sexual communication between mothers and daughters (Aronowitz & Morrison-Beedy, 2004). Among AA families, higher maternal connectedness has been shown to be associated with lower sexual risk behaviors (Hahm et al., 2006).

Sexual communication between a mother and daughter is an effective strategy to help delay the age of sexual initiation, promote sexual health, and reduce sexual risky behavior (Meneses, Orrell-Valente, Guendelman, Oman, & Irwin, 2006; Romo, Cruz, & Neilands, 2011). The earlier sexual initiation occurs the more likely the woman is to have multiple partners. Studies with minority youth such as African Americans and Latinas have shown the effectiveness of mother-daughter sexual communication as a significant protective factor to reduce sexual risky behavior in adolescence (Aronowitz, Ogunlade, Nwosu, & Gona, 2015; Villarruel, Cherry, Cabriales, Ronis, & Zhou, 2008).

However, communication about sex is rare in AA families because of the Asian cultural norm that discussing sex or sexual-related topics is extremely taboo (Okazaki, 2002). This taboo remains an issue today (Zhao et al., 2017). Seventy percent of AA youth reported they have never had sexual health related communication with their parents (Kim, 2009). AA mother-daughter dyads have higher levels of discomfort about sexual communication as compared to African American and Latina dyads (Meneses et al., 2006). This Asian cultural norm often remains in AA immigrant families that have been living in the U.S. Risky sexual behaviors that AA youth participate in has been closely associated with the degree of their acculturation (Hahm et al., 2012; Kao, Loveland-Cherry, Guthrie, & Caldwell, 2011; Schwartz et al., 2011).

**Figure 1.**
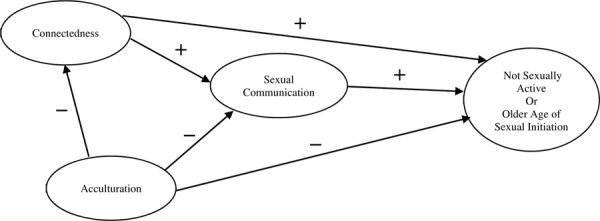
Predicted model of factors that affect sexual initiation among Asian American youth.

The purpose of this study was to examine whether mother-daughter connectedness and level of acculturation predicts sexual communication in turn affecting the age of their sexual initiation. Six hypotheses were tested: (1) Increased connectedness between AA mothers and daughters will delay daughters’ sexual initiation; (2) Increased acculturation of AA daughters leads to their earlier sexual initiation; (3) Increased open sexual communication between AA mothers and daughters will delay the daughters’ sexual initiation; (4) Increased AA daughters’ acculturation leads to less connectedness to their mothers; (5) Increased acculturation of AA daughters will lead to less sexual communication with their mothers; and (6) Increased connectedness between AA mothers and daughters will lead to open sexual communication. If bivariate analysis showed significant relationships among the variables, we would explore whether there was mediation effect of sexual communication (see Figure 1).

## Methods

### Design

The design of the study was a longitudinal secondary analysis of the National Longitudinal Study of Adolescent to Adult Health (Add Health) employing Wave I (1995) and Wave III (2002) of the in-home interview data. The Add Health study was funded by the National Institute of Child and Human Development and 17 other federal agencies (Bearman, Jones, & Udry, 1997). It was designed to examine the causes of health-related behaviors in adolescents in the U.S. It included a random national sample of youth, contains no identified information, and has been used extensively for secondary analysis. Because of the persistent taboo regarding sexual communication among AAs and the lack of studies examining this phenomenon in this population, Add Health (even though dated) is an important venue to examine this area of study in a longitudinal manner.

### Study Protocol

The inclusion criteria of this study were mothers and daughters from East Asia (China, Japan, Hong Kong, Korea, and Taiwan) and Southeast Asia (Cambodia, Vietnam, Thailand, and Philippines) (U.S. Census Bureau, 2018) descent within the Wave I data set. AAs from these regions have similar cultural values, attitudes, and behaviors (Dissanayake et al., 2015; Greenwood et al., 2016). Linked data of mothers and daughters from Wave III was included in the analysis. Asian Indian families from South Asia (India, Pakistan, and Nepal) (U.S. Census Bureau, 2018) were excluded because of the cultural differences. Because South Asia has been influenced more by Hinduism and Islam (Greenwood et al., 2016), we excluded the participants from these geographic areas. Institutional Review Board approval as exempt status was obtained from the researchers’ institution.

## Measures

### 

#### Sociodemographics

AA daughters’ age and place of birth were recorded at Wave I. The place of birth was measured by whether they were born in the U.S or not. The education levels for both mothers and daughters were measured at Wave III.

#### Acculturation

This concept was measured using four items (two 4-point Likert format and two dichotomous yes/no items) from Wave III of the data that were asked of the daughter. Wave III data was used since it allowed the daughters to answer questions about their own acculturation when they were emerging adults. Potential score range was 0–8, with higher score meaning they were more acculturated. Example questions included language that the daughter spoke at home and with friends and how often they read newspaper, bought music, and watch television in a language other than English. There were no acculturation questions for mother therefore only daughter’s acculturation was employed in the study. Questions that were included are similar to other instruments used to measure acculturation among Asian populations with Cronbach alphas ranging from 0.75 to 0.79 (Ryder et al., 2000).

#### Connectedness

This concept was measured using five items from Wave I reported by daughters, when they were high school age. Questions were 5-point Likert format. Potential score range was 5–25 with higher score meaning greater connectedness to mother. Example question included “how much do you think mother cares about you?” The same questions used in this study has been used in a previous study of the AddHealth data of early adolescent females (Aronowitz & Morrison-Beedy, 2004), with Cronbach alpha score of 0.87.

#### Sexual communication

This concept included 11 items from Wave I of the data, reported by mother. Five items were 5-point and six items were 4-point Likert format with a potential score range of 11–49, with higher score meaning more sexual communication. Example question included how much mother agree with “How much have you and your daughter talked about having sex and the dangers of getting a sexually transmitted infection (STI)?” These questions have been used in other studies measuring sexual communication among minority adolescents with a Cronbach alpha of 0.93 (Aronowitz, Rennells, & Todd, 2005; Lehr, Dilorio, Dudley, & Lipana, 2000).

#### Sexual initiation

The outcome variable was sexual activity (vaginal penetration), which was measured by daughters reporting: (1) having had sex or not and (2) their self-report of age of sexual initiation both measured in Wave III.

### Data Analysis

Descriptive analyses such as means, standard deviations, and frequencies were completed for all the variables. Correlation analyses were completed using Pearson’s correlation for continuous variables. Bivariate analyses such as *t*-tests and Chi-square tests were followed to evaluate sociodemographic and key concept relationships to sexual initiation. Multivariate linear regression analyses and path analyses were also completed to examine the relationship of the key concepts with sexual initiation. SAS software version 9.4 (SAS Institute Inc., Cary, NC) was used to complete the analyses. G*Power was calculated using significance level set as an a = 0.05, and power = 0.8 for sample size of 242.

## Results

### Sample

There were 243 Asian American mother-daughter dyads in Wave I with linked data to Wave III that were included in the study. At Wave I, the daughters’ age ranged from 12 to 19 years (*M* = 16 years; *SD* = 1.6) and mothers’ age ranged from 20 to 64 years (*M* = 44.9 years; *SD* = 6.7). Over half of the sample (60%) self-identified as Filipino. At Wave I, half of the sample (51%) reported that the daughters were foreign born. Almost half of the mothers (49.4%) reported having a graduate education. Since the data was linked, the sample was seven years older at Wave III (19–26 years). Eighty-eight percent of the daughters had completed four years of college and 10% had completed two years of graduate education.

#### Univariate

The mean score on acculturation reported by daughters at Wave III was *M* = 6.7 (*SD* = 1.5), Cronbach alpha = 0.66. Connectedness measured at Wave I reported by daughters had a *M* = 21.1 (*SD* = 3.8), Cronbach alpha = 0.87. Sexual communication was measured by mothers’ report in Wave I with a *M* = 35 (*SD* = 8.1), Cronbach alpha = 0.87. In Wave III, daughters’ reported age of sexual initiation was *M* = 17.4 years (*SD* = 2.2).

#### Bivariate

The bivariate analyses of the independent variables and their relationship to the interval level outcome variable of age of sexual initiation had two significant findings. The bivariate correlation between acculturation and age of sexual initiation was significant (*r* = −0.30, *p* < 0.001), supporting Hypothesis 2. Acculturation was positively associated with sexual communication (*r* = 0.26, *p* < 0.001), which is opposite to Hypothesis 5. Connectedness was not significantly related to either acculturation (*r* = −0.04, *p* = 0.52), sexual communication (*r* = −0.10, *p* = 0.12), or age of sexual initiation (*r* = 0.04, *p* = 0.60), thereby not supporting Hypotheses 1 or 4.

In the bivariate analyses, the dichotomous outcome variable had sex or not was significantly correlated to the outcome. The association with sociodemographic characteristics and the independent variables were examined using *t*-tests or Chi-square tests (see Table 1). We found the higher the level of sexual communication with mothers the greater likelihood for daughters to report having been sexually active, not supporting Hypothesis 3. They were also more likely to have graduated from high school.

**Table 1:** Sociodemographics and Independent Variables Affecting Sexual Initiation

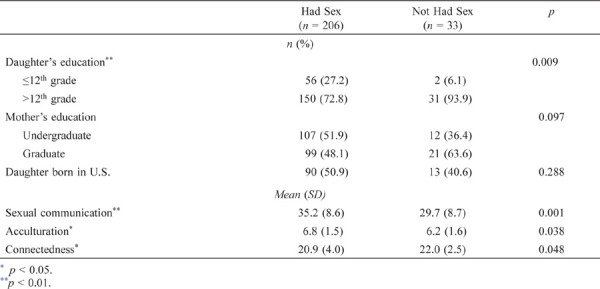

**Table 2:** Logistic Regression of Factors that Affect Sexual Initiation (Had Sex or Not)

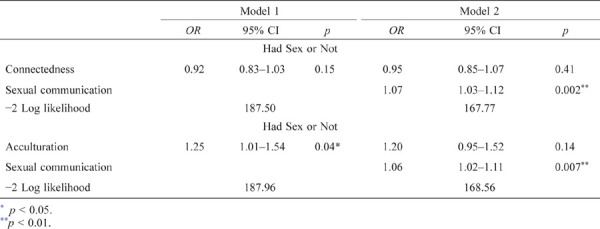

**Table 3:** Multivariate Linear Regression for Age of Sexual Initiation

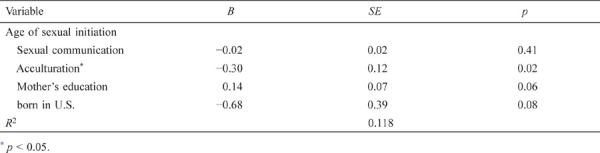

#### Multivariate

Exploratory analyses were completed to further examine the significant associations among acculturation, connectedness, sexual communication, and sexual initiation, controlling for sociodemographic characteristics. Logistic regression analyses were examined using the concepts that were found to have significant bivariate relationships (see Table 2). Model 1 explored the relationship between sexual communication and having sex or not controlling for connectedness, and Model 2 explored this relationship controlling for acculturation. The results showed that connectedness was not a significant factor to predict sexual initiation. AA daughters who were more acculturated were 25% more likely to have initiated sexual activity. However, once sexual communication was added to the model, the association between acculturation and had sex or not was no longer significant. AA daughters who have more open sexual communication with mothers had six to seven percent higher risk of having had sex, compared to those with less sexual communication. Sexual communication may play a mediation role in the relationship between acculturation and sexual initiation. When using age of initiation as the outcome variable, the multivariate linear regression analyses showed that acculturation was significant (b = −0.30, *p* = 0.02), after controlling for AA mothers’ education and daughters’ place of birth (see Table 3). Sexual communication was not significantly associated with age of sexual initiation (*p* = 0.41). The *R*2 for the full-adjusted model was 0.118.

## Discussion

For this sample of AA females, the mean age of sexual initiation (*M* = 17.4; *SD* = 2.2) was similar to the U.S. average (Finer & Philbin, 2014), and comparable to other studies of AA youth (Cavazos-Rehg et al., 2009; Hahm et al., 2006). The majority of AA daughters had completed a four-year college by Wave III. AA mothers were also well-educated, with 50% having completed graduate education. Filipinos were the most prevalent population in this data set, which can be understood due to the trends of Southeast Asian immigration from the end of World War II into the 1990s. During that time, the Filipino population’s cultural values were congruent with other East and Southeast Asian countries (Boyd, 1971; Obligacion & Obligacion-Arboleda, 1994).

The greater acceptance of American culture the daughters had, the more likely they were to follow the social norms of their peers (Finer & Philbin, 2014) and initiate sexual activity at an earlier age. However, opposite to Hypothesis 5, the higher level of acculturation the daughters had, the more likely they were to report having open sexual communication with their mothers. It is difficult to make a conclusion about the directionality of this relationship because this was a secondary analysis research design and the research team had to formulate the measure of acculturation from the items available within the dataset. It is likely that acculturation is multi-dimensional and was not fully operationalized by the items available in the data set.

According to the results of multivariate analyses, acculturation was found to only indirectly affect sexual initiation through sexual communication. The way acculturation was measured (only by language use) could affect sexual communication especially for AA mothers who were not acculturated and used their heritage language. They may not have had the words to discuss sexual health with their daughters. The high level of education in both mothers and daughters’ in this sample did not have an effect on their sexual communication. This may be because no matter how long the mothers live in the U.S., they may maintain their Asian heritage values of feeling that sexual communication is taboo (Kim, 2009; Yu, 2007). It is possible that the influence of Asian values may increase the mothers’ discomfort and have a stronger effect on communication than their level of academic education. However, due to the unexpected finding of the relationship between sexual communication and earlier sexual initiation, it is unclear if the communication occurred before or after the daughters initiated sexual activities. Future studies should use a more complete definition of acculturation to see if there is a direct effect of acculturation on sexual initiation. Furthermore, it would be important to identify the AA mothers’ level of acculturation and to understand whether there is any interaction between the dyads’ level of acculturation that might create an intergenerational gap.

There was no direct or indirect effect of connectedness between AA mothers and daughters on daughters’ sexual initiation. Although connectedness has been found to be important in postponing sexual initiation and decreasing risk behaviors among other minority youth (Aronowitz et al., 2015; Villarruel et al., 2008), this was not observed in this sample of AA youth. In another study with a sample of minority youth groups including AA youth, a relationship was found between connectedness and later sexual initiation in African American and Latino youth, but not in AA families (Gillmore, Chen, Haas, Kopak, & Robillard, 2011). Further exploration is needed to better understand how AA mothers and daughters define a connected relationship. One of the explanations may be that sexual health is such a taboo topic that even when mothers and daughters are closely connected, they still cannot have open sexual communication.

An interesting finding was that the greater level of sexual communication that the mother reported, the more likely the daughter was to report having initiated sexual activity. Sexual communication was only reported by mothers at Wave I, which was when the daughters were between 12 and 19 years old. Since the age of the sexual initiation in the sample was 17 years of age, it is unclear whether the mothers initiated the communication because of the concerns of their daughters’ peer relationships or as a means of promoting sexual health. In other words, mothers may have initiated this communication after suspicion of daughters’ sexual activity as has been suggested in a previous study (Somers & Paulson, 2000). Therefore, further research is needed not only to question what topics they discussed, but when the sexual communication occurred. Additionally, a dyadic approach to measuring open sexual communication could assure that there is no discordance between mothers’ and daughters’ interpretation of the communication.

## Limitations

As with any secondary analysis study, there are limitations due to the preexisted dataset and limited items within the dataset to operationally define the concepts of interest. Although Wave I and III used for the study are dated, there is scant data on AA daughter’s sexual health and this study provides a seven-year longitudinal examination of this phenomenon. Because of the strong Asian cultural values surrounding issues related to sexual health, things have not changed in the past two decades (Tong, 2013; Zhao et al., 2017). The multivariate regression analysis accounted for only 12% of variance in the model, therefore further research is needed to explore potential other variables that affect sexual initiation in this population.

## Conclusion

Further studies should focus on a dyadic approach to sexual communication, to include not only the specific topics but both individuals within the dyads’ perception of what was communicated. Measures of sexual communication need to identify the topics of the discussion and the timing of that conversation in relation to sexual initiation. To best transmit parental values, their open sexual communication needs to occur prior to sexual activity. Although acculturation was found to be a significant predictor of sexual behavior, the concept needs to be measured with a dyadic approach to better understand how acculturation affects the mother–daughter relationship. Acculturation measurement needs to include various categories other than language use. Formative research is needed to better define connectedness and understand the relationship between connectedness and youth risk behaviors in AA families.

## Acknowledgments

This research uses data from Add Health, a program project designed by J. Richard Udry, Peter S. Bearman, and Kathleen Mullan Harris.

## Declaration of Conflicting Interests

The authors declared no potential conflicts of interest with respect to the research, authorship, and/or publication of this article.

## Funding

This research was funded by grant P01-HD31921 from the National Institute of Child Health and Human Development, with cooperative funding from 17 other agencies.
